# The hidden hand of chloride in hypertension

**DOI:** 10.1007/s00424-015-1690-8

**Published:** 2015-01-27

**Authors:** Linsay McCallum, Stefanie Lip, Sandosh Padmanabhan

**Affiliations:** BHF Glasgow Cardiovascular Research Centre, Institute of Cardiovascular and Medical Sciences, University of Glasgow, Glasgow, G12 8TA UK

**Keywords:** Salt, Chloride, Hypertension, Blood pressure, Anion

## Abstract

Among the environmental factors that affect blood pressure, dietary sodium chloride has been studied the most, and there is general consensus that increased sodium chloride intake increases blood pressure. There is accruing evidence that chloride may have a role in blood pressure regulation which may perhaps be even more important than that of Na^+^. Though more than 85 % of Na^+^ is consumed as sodium chloride, there is evidence that Na^+^ and Cl^−^ concentrations do not go necessarily hand in hand since they may originate from different sources. Hence, elucidating the role of Cl^−^ as an independent player in blood pressure regulation will have clinical and public health implications in addition to advancing our understanding of electrolyte-mediated blood pressure regulation. In this review, we describe the evidence that support an independent role for Cl^−^ on hypertension and cardiovascular health.

Essential hypertension is the result of a complex interplay between multiple regulatory systems which are themselves influenced by a multitude of genetic and environmental factors. Among the environmental factors that affect blood pressure, dietary sodium chloride has been studied the most, and there is general consensus that increased sodium chloride intake increases blood pressure. The role for NaCl is supported by insights from the pressure-natriuresis mechanism [30], monogenic forms of hypertension [51], and dietary salt reduction studies [16, 32, 76]. However, there is still considerable debate about NaCl and hypertension particularly in relation to the context in which this occurs, its prognostic implications, and the role of the underlying regulatory and counter-regulatory pathways that are perturbed when salt intake is altered [2, 25, 43, 60, 62, 65–67]. The blood pressure response to sodium chloride intake is referred to as salt sensitivity and while this has universal definition, a 5–10 % change in office blood pressure in response to a change in salt intake is indicative. Importantly, studies of salt sensitivity show that the blood pressure responses to salt are variable and demonstrate a Gaussian distribution within populations. Salt sensitivity is more prevalent in hypertensive individuals (30–50 %) compared to normotensives, and the presence of salt sensitivity in normotensives is a risk factor for future development of hypertension [95]. Salt sensitivity is not specifically NaCl related, as it can be modulated by other components of the diet including potassium, calcium, protein, carbohydrate, and fat [45, 53]. There is growing evidence that Cl^−^ component of NaCl may have a more specific role in salt-sensitive blood pressure, and this may perhaps be even more important than that of Na^+^. But this is neither a recent nor novel idea; a role for Cl^−^ had been mooted as early as 1904 by Ambard and Beaujard [3] and in 1908 by Higgins [34] who pointed out that hypertension was intimately associated with “chlorine retention.” Then in 1929, Berghoff and Geraci [5] noted that loading hypertensive individuals with sodium bicarbonate did not have the same pressor effect as loading with sodium chloride. Since then, an independent effect of Cl^−^ has been re-discovered in the 80s using diet containing citrate or phosphate as the anion for Na^+^ [48, 49, 83, 97], and again more recently from epidemiologic outcome studies showing contrasting associations of serum Cl^−^ and Na^+^ on mortality [19, 59]. In usual diets, more than 85 % of Na^+^ is consumed as sodium chloride and any clinical relevance of the independent effect of Cl^−^ on blood pressure and prognosis has been considered to be largely “academic” [39]. However, studies measuring Na^+^ and Cl^−^ content in processed foods indicate that Na^+^ and Cl^−^ concentrations do not go necessarily hand in hand since they may originate from different sources [13]. In this review, we shall describe the role of Cl^−^ as a significant electrolyte in its own right with major impact on hypertension and health.

## Dietary chloride and blood pressure—animal and human studies

There is data from three experimental rat models (Dahl salt-sensitive rat, DOCA salt-sensitive rat, and SHRSP) demonstrating that the full expression of NaCl-dependent hypertension is reliant on the concomitant provision of both Na^+^ and Cl^−^. Early studies in Dahl salt-sensitive rats [1, 44, 46] showed that hypertension occurred within several weeks when animals were fed on a high NaCl diet, but not when the animals were fed an identical Na^+^ load provided as sodium bicarbonate or other non-chloride salts of Na^+^. Kurtz and Morris [49] showed similar findings in a model of desoxycorticosterone acetate (DOCA) salt-sensitive hypertension where after administration of DOC, the mean systolic blood pressure in rats given sodium chloride were significantly higher than in those given sodium bicarbonate and/or sodium ascorbate. The failure of selective dietary sodium loading to produce hypertension was not related to differences of body weight, net Na^+^ balance, blood pH, serum concentrations of Na^+^, potassium, or chloride [1, 44, 46, 49]. Passmore and Jiminez [73] showed that in DOCA salt-sensitive hypertensive rats, blood pressure and renal vascular resistance were significantly higher in rats consuming a diet high in Cl^−^ while cardiac output was related to Na^+^ intake. Their group followed this with studies showing that the pressure-flow curves of the DOCA-high Cl^−^ groups shifted significantly downward (reduced renal blood flow at all pressures) and rightward (elevated lower threshold) compared with the DOCA-normal NaCl and -high Na^+^ groups [37]. Luft et al. [54] reported that supplementation of NaCl in drinking water caused a modest but significant increase of arterial pressure in the stroke-prone spontaneously hypertensive rat (SHRSP), whereas an equivalent Na^+^ load, primarily in the form of sodium bicarbonate, did not. The increase in blood pressure with Cl^−^ alone was less than the increase with NaCl in both Dahl salt-sensitive rat and SHRSP, suggesting that the pressor “sensitivity” to dietary NaCl depends on both its Na^+^ and Cl^−^ components [96, 100]. Schmidlin et al. [77] and Tanaka et al. [89] in a study of SHRSP showed that dietary Cl^−^ was selectively sufficient to induce a pressor effect and that the Na^+^ component of dietary NaCl was not selectively sufficient to induce a pressor effect. Further support for the role of Cl^−^ comes from the renin response to Na^+^ and Cl^−^. The rise in blood pressure with NaCl intake is usually accompanied by suppression of the renin-angiotensin system and decreased plasma renin. Acute and chronic administration of non-chloride sodium salts did not appear to suppress plasma renin activity in rats, while renin was inhibited by both sodium chloride and by selective Cl^−^ (without Na^+^) loading both in rats and humans [1, 38, 44, 46]. Curiously, a diet containing a combination of sodium iodide and sodium bromide induces hypertension more readily than other non-chloride sodium salts in DOCA-treated rats, suggesting that the role of Cl^−^ in the effect of NaCl on blood pressure may be related to some property common to halides [7].

A limited number of clinical observations also suggest that blood pressure is not increased in humans by high dietary Na^+^ intake in the absence of Cl^−^. The earliest clinical study in 1929 showed that Cl^−^ was the main blood pressure increasing component with the observation that sodium bicarbonate did not have the same pressor effect as sodium chloride in hypertensive individuals [5]. Kurtz et al. [48] demonstrated that the rise in blood pressure in response to a high sodium diet (high-salt diet, 240 mmol sodium chloride per day; 5.52 g Na^+^) was abolished upon substituting an equimolar amount of sodium citrate. Luft et al. [55] showed opposite effects of NaCl and NaHCO_3_ on blood pressure and calcium excretion with NaHCO_3_ reducing blood pressure but increasing calcium excretion. Shore et al. [83] reported that NaCl intake induced a greater rise in blood pressure than sodium phosphate intake. In 1945, Grollman showed that both Na^+^ and Cl^−^ were required to increase blood pressure in humans when dietary supplementation with ammonium chloride failed to increase blood pressure of hypertensive humans after dietary NaCl restriction had decreased blood pressure [28]. Also, in hypertensive humans, the reduction of blood pressure by dietary potassium was attenuated by potassium chloride compared with that of potassium citrate [71], but this difference was not observed in other studies [8, 33], and supplemental potassium chloride did not reduce the need for antihypertensive medication in hypertensive men on a restricted-sodium diet [27].

## Serum chloride and outcomes

Large epidemiologic studies curiously show that lower circulating levels of Cl^−^ are associated with higher cardiovascular and all-cause mortality. De Bacquer et al. [19] studied 9106 participants from the Belgian Interuniversity Research on Nutrition and Health (BIRNH) study who were followed up for 10 years. They showed serum Cl^−^ <100 mEq/L was associated with an increased risk of all-cause, cardiovascular disease, non-cardiovascular disease, and coronary heart disease mortality after adjustment for age, body mass index (BMI), and serum Na^+^ levels. Serum Cl^−^ <100 mmol/L was found to be a strong predictor (RR 1.77; 95 % CI 1.22–2.5), in multivariate analysis, of total, cardiovascular, and non-cardiovascular mortality independent of other classic risk factors and larger than the effects of diabetes (RR 1.46; 95 % CI 0.81–2.63), smoking status (RR 1.50; 95 % CI 1.10–2.05), BMI (RR 1.20; 95 % CI 0.85–1.67), and cholesterol levels (RR 1.16; 95 % CI 0.88–1.54).

A post hoc analysis of the Candesartan in Heart failure-Assessment of Reduction in Mortality and Morbidity (CHARM) study [21] aimed to identify novel prognostic markers in heart failure in a cohort of 2679 American patients. The multivariate analysis showed that serum Cl^−^ was a predictor of all-cause mortality in patients with heart failure with an adjusted HR 0.78 per SD increase in serum Cl^−^ (95 % CI 0.71–0.85), suggesting that serum Cl^−^ predicts risk independently of blood pressure and serum Na^+^.

A large study of 12,968 treated hypertensive patients attending the Glasgow Blood Pressure Clinic with a follow up period of 197,101 person-years sought to investigate the association between serum Cl^−^ and mortality [59]. Similar to De Bacquer et al. [19], this study reported that individuals with serum Cl^−^ <100 mEq/L had the lowest survival independent of serum Na^+^ or HCO_3_^−^ levels (*p* < 0.001). Multivariate, adjusted, analysis showed an inverse association between serum Cl^−^ and all-cause mortality; a 1.5 % reduction in all-cause mortality is seen for every 1 mEq/L increase in serum Cl^−^ (HR 0.985; 95 % CI 0.980–0.990). Similar results were shown for cardiovascular disease mortality (HR 0.985; 95 % CI 0.978–0.991), ischaemic heart disease mortality (HR 0.985; 95 % CI 0.976–0.995), and non-cardiovascular disease mortality (HR 0.985; 95 % CI 0.977–0.990). The association with stroke mortality did not reach statistical significance (HR 0.996; 95 % CI 0.981–1.010).

The mechanism by which low serum Cl^–^ increases mortality or cardiovascular events is unclear [23]. The risk associated with low serum Cl^–^ appears to be independent of serum Na^+^, K^+^, or anion gap [59]. This also suggests that dietary Cl^–^ and serum Cl^–^ exert different effects and perhaps the regulation of serum Cl^–^ is not entirely related to dietary intake and renal mechanisms. Emerging evidence that the immune system plays an extrarenal regulatory role in Na^+^ homeostasis and the intriguing finding that when this immune mechanism was blocked there was selective Cl^−^ accumulation in the skin salt-sensitive hypertension would support this hypothesis [56, 57, 98].

## Chloride physiology

Cl^−^ is the principal extracellular and intracellular anion in the body representing 70 % of the total negative ion content and about 0.15 % of total body weight (115 g in an adult). The normal plasma concentration of Na^+^ is 135–145 mEq/L. Because of its high concentration, Cl^−^ is critical in maintaining electroneutrality. Cl^−^ is responsible for about 100 of the 300 mOsml/L of extracellular fluid tonicity [4]. Regulatory mechanisms for volume homeostasis are generally triggered by changes in Na^+^ and Cl^−^ concentrations. Cl^−^ has an inverse relationship with bicarbonate and this maintains acid-base balance through reciprocal transport into and out of erythrocytes and renal tubuli [4]. Cl^−^ excretion is an important mechanism in the kidney’s adaptation to metabolic acidosis and chronic respiratory acid-base disturbances. Circulating Cl^−^ concentrations are mainly regulated by the gastrointestinal tract and the kidneys. Cl^−^ is absorbed along the entire length of the intestine, secreted by the gastric parietal cells as HCl. The driving flux for fluid secretion into the intestinal tract is the osmotic gradient between the intestinal lumen and the mucosa which is mainly generated by Cl^−^ and to a lesser extent by HCO_3_^−^. Normally, the kidney adapts urinary Na^+^ and Cl^−^ excretion to match exactly daily dietary NaCl intake. Cl^−^ channels are expressed along the entire mammalian nephron and participate in transepithelial Cl^−^ transport, cell volume regulation, and acidification of intracellular vesicles [92]. Renal reabsorption of Na^+^ and Cl^−^ is tightly linked in most segments, often occurring even through the same transport proteins such as the Na^+^-K^+^-2Cl^−^ cotransporter NKCC_2_ or the Na^+^-Cl^−^ cotransporter NCC in the thick ascending limb or the distal tubule, respectively. In the proximal tubule and in parts of the collecting system, the transport of Cl^−^ and Na^+^ is mediated by separate mechanisms and Cl^−^ fluxes occur through both paracellular and transcellular routes. Remarkably, in the connecting tubule (CNT) and the collecting duct (CD), Na^+^ reabsorption is not linked molecularly to Cl^−^ transport directly but appears to be linked to bicarbonate secretion. In the collecting duct, particularly in the CNT and cortical collecting duct (CCD), Na^+^ is reabsorbed via the aldosterone-sensitive luminal epithelial Na^+^ channel (ENaC) and the basolateral Na^+^-K^+^-ATPase in principal cells. However, these cells appear to have almost no Cl^−^ conductance on both membranes, excluding them as the route for transcellular Cl^−^ transport. In contrast, neighboring intercalated cells express a number of anion transport and anion channel proteins. The Na^+^-independent Cl^−^/HCO_3_^−^ exchanger, pendrin (SLC26A4), is located on the apical membrane of B-intercalated cells in the kidney DCT, CCD, and the CNT and mediates the secretion of HCO_3_^−^ and the reabsorption of Cl^−^. Type A intercalated cells in contrast express the H^+^-ATPase on the apical plasma membrane and the Cl^−^/HCO_3_^−^ exchanger (AE1) on the basolateral plasma membrane. Pendrin may be regulated by the urinary excretion of Cl^−^. With depleted urinary Cl^−^, pendrin is upregulated and when large amounts of Cl^−^ are delivered to the CCT, the expression of pendrin is reduced [12, 93]. The basolaterally expressed Cl^−^/HCO3^−^ anion exchanger, AE1, that releases bicarbonate into blood belongs to a subfamily of electroneutral anion exchangers of the SLC4 family of bicarbonate transporters. AE1 is abundant in the red cell membrane, where it is an integral part of the cell’s cytoskeleton where it has a key role in the normal gas transfer of CO_2_. It is also expressed in the basolateral membrane of the collecting duct acid-secreting cell, though as a shorter N-terminally truncated form (kAE1), where it transports intracellular HCO3^−^ out of the cell in exchange for Cl-. Mutations in AE1 cause distal renal tubular acidosis, hereditary spherocytosis, and Southeast Asian ovalocytosis [86, 99].

The intracellular concentration of Cl^−^ is much lower than its plasma concentration and depends on the resting membrane potential of the cell and ranges from 2–4 mEq/L in muscle cells and 100–120 mEq/L in smooth muscle cells and red blood cells [4, 29]. The Cl^−^ channels in other tissues include (1) the ClC family of Cl^−^ channels that are often voltage-gated, (2) the cystic fibrosis transmembrane conductance regulator (CFTR), a member of the ABC transporter family, (3) the ligand-gated GABA and glycine-activated Cl^−^ channels, (4) the calcium-activated Cl^−^ channels and bestrophins, and (5) the transmembrane protein 16 (TMEM16)/anoctamin (ANO) [20, 92].

## Chloride, blood pressure, and cardiovascular risk—putative mechanisms

A direct role for Cl^−^ on hypertension is not established currently. However, evidence from monogenic syndromes, dietary and animal studies on renal Cl^−^ balance, and Cl^−^ transporters in vascular tissues point to a critical role for Cl^−^ in mechanisms that contribute to blood pressure regulation.

Monogenic syndromes associated with Cl^−^ transporters manifest high and low blood pressure phenotypes. In Gordon’s syndrome (pseudohypoaldosteronism type II), hypertension occurs as a consequence of increased Cl^−^ reabsorption in the thiazide-sensitive segment of the distal renal tubule [87]. Bartter syndrome is associated with salt wasting, hypokalemia, metabolic alkalosis, and increased renin secretion and is caused by inactivating mutations in genes encoding ion channels and transporters that mediate salt transport in the thick ascending limb of the loop of Henle [6]. The genes implicated in Bartter’s syndrome are the NKCC2, the potassium channel (ROMK), one of the Cl^−^ channels (CIC-Ka), and barttin (an essential subunit for the Cl^−^ channels CIC-Ka and CIC-Kb).

Extensive investigations in several models of hypertensive rats and in humans show that loading with equimolar amounts of sodium salts causes similar degrees of Na^+^ retention, weight gain, and suppression of RAAS, but only sodium chloride causes an expansion of plasma volume and a rise in BP [7]. In Dahl-S rats, DOCA salt rats, and in humans, plasma volume is higher on a high NaCl intake than when Na^+^ is provided with anions other than Cl^−^, although net Na^+^ balances do not differ. This suggests that the anion ingested with Na^+^ affects the distribution of Na^+^ between the intracellular and extracellular compartments [7].

Cl^−^ reabsorption in the cortical segment of the loop of Henle is greater in Dahl-S than in Dahl salt-resistant (R) rats when both are examined at equivalent renal perfusion pressures [40]. This finding is present before exposure to a high NaCl diet and before the onset of hypertension. Enhanced reabsorption of water and Cl^−^ in the loop of Henle may contribute to the blunted natriuretic capacity and hence to hypertension in Dahl-S rats. In the Dahl-S rat, if Cl^−^ delivery to the loop is related to dietary Cl^−^ intake, decreased renal tubular reabsorption of Cl^−^ may account for the failure of non-chloride salts of Na^+^ to increase blood pressure [42].

In vivo, in isolated perfused kidneys, and in kidneys perfused in situ, hyperchloremia results in renal vasoconstriction and a decline in glomerular filtration rate as a consequence of tubuloglomerular feedback [77, 78]. This suggest that tubuloglomerular feedback is activated by increased Cl^−^ delivery to the macula densa in chloride-fed animals, resulting in increased renal afferent arteriolar resistance, reduced renal blood flow and glomerular filtration rate, and increased systemic arterial pressure.

In clinical studies, the failure of blood pressure to fall significantly during the non-chloride Na^+^ salt phase may reflect the fact that the enrolled subjects in most of these studies were primarily salt-sensitive. A high dietary sodium chloride intake has been shown to increase the pressor response to both norepinephrine [75] and angiotensin II [36] and thus contribute to salt sensitivity. However, Sharma et al. [81] showed that the pressor response to norepinephrine and angiotensin II is dependent on Na^+^ but not on Cl^−^, and this finding in conjunction with the observation that blood pressure increased with NaCl but not with non-halide Na^+^ would suggest that enhanced pressor response is not the sole mechanism responsible for salt sensitivity [81, 82].

The regulation of release and synthesis of renin by the juxtaglomerular cells in response to body salt content is multifactorial and involves angiotensin II, autacoids released from endothelial or macula densa (MD) cells, various hormones, and the intraluminal blood pressure in afferent arterioles [47]. The MD mechanism for control of renin secretion is through tubular salt sensing, and a reduced NaCl concentration in the macula densa segment of the nephron elicits an activation of the renin-angiotensin system. There is ample evidence that the MD cells senses luminal NaCl concentration via the NKCC2 cotransporter and that a reduction in NaCl concentration results in stimulation of renin release and renin synthesis [69, 84]. There is considerable evidence that renin release is inhibited by increased Cl^−^ delivery to the macula densa or increased Cl^−^ transport across the thick ascending limb of the loop of Henle [52, 80]. Cl^−^ dependence has been related to the involvement of NKCC2 in the initiation of a transmitted MD signal, and the relative affinities of NKCC2 for Na^+^ and Cl^−^ are such that the Cl^−^ ion is predicted to act as the dominant physiological regulator of NKCC2 transport rate, and this mechanism is virtually inoperative when luminal Cl^−^ is low [10, 26, 79]. In studies of uromodulin knock-out mice, Mutig et al. [63] showed that activation of NKCC2 is facilitated by uromodulin in a Cl^−^-sensitive manner, which is interesting as there is now an accrual of data from human genome-wide association studies and rodent studies that uromodulin is associated with hypertension and salt sensitivity [24, 72, 90]. The release of prostaglandin E2 from the macula densa and the adjacent thick ascending limb of Henle’s loop increases when the concentration of sodium chloride in the tubular fluid falls [74, 103]. Such a fall in tubular NaCl concentration at the macula densa site is thought to occur in states of salt deficiency and in situations of reduced glomerular filtration. The data from salt-restricted humans does not show difference in renin and angiotensin II production with NaCl and non-halide Na^+^ dietary intake [22, 48, 55, 81–83], while the phenomenon is not as consistently observed in rodents and may suggest species differences in BP regulation [46, 52].

Several early studies demonstrated Cl^−^ flux in a variety of different vascular smooth muscle cell (SMC) types. Noradrenaline stimulated Cl^−^ efflux in rat aorta [85] and rabbit pulmonary veins [14]. Endothelin activates Cl^−^ currents in porcine coronary artery, human mesenteric artery SMCs, [41], and cultured aortic SMCs [91]. Studies using non-selective Cl^−^ channel antagonists [64, 88] and anion replacement [17, 50, 94] support the concept that Cl^−^ flux contributes to vasoconstriction. 4,4′-Diisothiocyanatostilbene-2,2′-disulfonic acid (DIDS) and indaryloxyacetic acid (IAA-94) hyperpolarized and relaxed pressurized rat cerebral arteries [64]. IAA-94 inhibited ET-induced vasoconstriction in cultured vascular SMCs [88]. Lowering extracellular Cl^−^ potentiated pressure-induced constriction in rat cerebral arteries [64]. In addition to modulating SMC contractility, both volume-sensitive Cl^−^ channels and Ca^2+^-activated Cl^−^ channels have been proposed to control SMC proliferation [15, 101]. ClC-3 has been found to be ubiquitously expressed in almost all eukaryotic cells, which functions as anion channel at cell plasma membrane or as Cl^−^/H^+^ antiporter in intracellular vesicles. Studies in vascular smooth muscle cell showed that several cytokines, including tumor necrosis factor-α (TNFα) and interleukin 1β, could activate Cl^−^ conductance, and this Cl^−^ current is dependent on ClC-3 expression [58]. ClC-3-dependent Cl^−^ efflux decreased intracellular Cl^−^, which underlies the proinflammatory effects of ClC-3-dependent Cl^−^ conductance by activating the NF-κB pathway [61, 102].

The ubiquitous Na^+^, K^+^, 2Cl^−^ cotransporter (NKCC1) belongs to the superfamily of Cl^−^-coupled carriers and are inhibited by high-ceiling diuretics such as furosemide and bumetanide. In the VSMC, loop diuretics decrease the concentration of intracellular chloride, hyperpolarize the sarcolemma, and attenuate Ca^2+^ influx though voltage-gated channels, indicating a putative mechanism by which NKCC1 contributes to hypertension via elevation of vascular tone [68, 70]. NKCC1-null mice have decreased baseline BP but exhibit augmented BP increment evoked by high-salt diets. NKCC1 deficiency causes approximately threefold elevation in plasma renin concentrations and attenuates high-ceiling diuretics-induced renin production [68, 70].

There is evidence of extrarenal regulatory mechanisms for electrolyte homeostasis with the finding of Na^+^ and Cl^−^ sequestration in the skin interstitium which appear to be regulated by the mononuclear phagocyte system [56, 57, 98]. Macrophages infiltrate to the sites of Na^+^ and Cl^−^ overload in the skin which display a hypertonic microenvironment and subsequently upregulate the transcription factor nuclear factor of activated T cells 5 (NFAT5) [31]. The induction of NFAT5 in macrophages of the skin was shown to directly govern the expression of vascular endothelial growth factor C (VEGF-C), resulting in the hyperplasia of lymph capillaries via and interaction with the VEGF receptor 3 (VEGFR3) [98]. Failure of this local extrarenal macrophage-dependent control mechanism to regulate interstitial electrolyte and water homeostasis resulted in arterial hypertension and massive disturbances in skin electrolyte composition [98]. Moreover, when NFAT5/VEGF-C axis was knocked out in experimental models, there was selective Cl^−^ accumulation in the skin, a direct correlation between skin Cl^−^ content and blood pressure increases, and no relationship between Na^+^ and water content and blood pressure [98].

The paradoxical association of serum Cl^−^ on mortality and the association of dietary Cl^−^ with blood pressure are intriguing and may help attempts to understand the role of Cl^−^ currents and osmolarity/volume homeostatic mechanisms. Gąsowski and Cwynar [23] hypothesize that low serum Cl^−^, whether associated with a hypoosmotic state or not, may facilitate Cl^−^ currents, acting as an enhancer to the phenomena that have been traced as possible triggers increasing the probability of an open state of these channels. Such phenomena include ischemia-induced local hypoosmotic state leading, in turn, to swelling of the cell and stimulation by tumor necrosis factor-α and interleukin-1β [18, 23, 35, 102]. Experimental evidence support the potential role of several Cl^−^ channels in the heart including CFTR, ClC-2, ClC-3, CLCA, Bestrophin, and TMEM16A which may contribute to cardiac arrhythmogenesis, myocardial hypertrophy and heart failure, and cardioprotection against ischemia–reperfusion [20]. Other potential mechanisms may relate to non-cardiac and non-renal roles for Cl^−^. For examples, Cl^−^ channels are present in the surface and transverse tubular membranes of mammalian skeletal muscle and Cl^−^ moves into muscle during t-tubular action potentials or with K^+^-induced depolarization of the sarcolemma. Extracellular Cl^−^ has been shown to be protective against fatigue (with implications for survival and cardiovascular risk) involving high-intensity contractions in both fast- and slow-twitch mammalian muscle possibly by preventing excessive depolarisation with exercise-induced decline in trans-sarcolemmal K^+^ gradient [9, 11].

In conclusion, Cl^−^-dependent mechanisms appear to underlie a plethora of critical pathways underlying cardiovascular disease and blood pressure regulation highlighting the need for further studies to elucidate the mechanistic underpinnings of these observations. However, the relationship between dietary chloride, serum chloride, and intracellular chloride all appear to have different pathophysiological effects, and further studies are needed to determine the mechanistic underpinnings of the epidemiologic findings. The weight of evidence indicate that it is time Cl^−^ moved out the shadow of Na^+^ as a mediator of disease and survival.
